# Achieving High Yield Strength and Ductility in As-Extruded Mg-0.5Sr Alloy by High Mn–Alloying

**DOI:** 10.3390/ma13184176

**Published:** 2020-09-19

**Authors:** Shibo Zhou, Xiongjiangchuan He, Peng Peng, Tingting Liu, Guangmin Sheng, Aitao Tang, Fusheng Pan

**Affiliations:** 1 College of Materials Science and Engineering, Chongqing University, Chongqing 400044, China; zhoushibo1994@foxmail.com (S.Z.); hxjc1993@foxmail.com (X.H.); gmsheng@cqu.edu.cn (G.S.); tat@cqu.edu.cn (A.T.); fspan@cqu.edu.cn (F.P.); 2 School of Metallurgy and Materials Engineering, Chongqing University of Science and Technology, Chongqing 401331, China; peng_pp@foxmail.com; 3 National Engineering Research Center for Magnesium Alloys, Chongqing University, Chongqing 400044, China; 4 School of Materials and Energy, Southwest University, Chongqing 400715, China

**Keywords:** Mg alloys, extrusion, microstructure, mechanical properties

## Abstract

The effect of Mn on the microstructure and mechanical properties of as-extruded Mg-0.5Sr alloy were discussed in this work. The results showed that high Mn alloying (2 wt.%) could significantly improve the mechanical properties of the alloys, namely, the tensile and compressive yield strength. The grain size of as-extruded Mg-0.5Sr alloys significantly was refined from 2.78 μm to 1.15 μm due to the pinning effect by fine α-Mn precipitates during the extrusion. Moreover, it also showed that the tensile yield strength and the compressive yield strength of Mg-0.5Sr-2Mn alloy were 32 and 40 percent age higher than those of Mg-0.5Sr alloy, respectively. Moreover, the strain hardening behaviors of the Mg-0.5Sr-2Mn alloy were discussed, which proved that a large number of small grains and texture have an important role in improving mechanical properties.

## 1. Introduction

Magnesium (Mg) alloys have the advantages of lightweight, outstanding specific stiffness, great specific strength, and dimensional stability, which lead to their wide application in aerospace, electronics, and transportation [1,2,3]. However, the poor deformation ability of Mg alloys at room temperature limits their large-scale application. This is mainly due to the fact that the Mg alloys with hexagonal closepacked (hcp) structure have less slip system at room temperature. Thus, in order to enhance the mechanical properties of extruded Mg alloys, massive studies have been done at home and abroad [4].

Some studies have been developed to improve the properties of extruded Mg alloys, such as weakening the texture to improve ductility [5,6,7], refining the microstructure to improve strength and ductility simultaneously [8,9,10,11,12], and implementing precipitation hardening to improve strength [2,13,14]. The mechanical properties of Mg alloys can be significantly improved by adding alloying elements. As we know, rare earth elements (REs) such as Ce, Y, Gd, and Nd can significantly improve the ductility of Mg alloys by weakening the texture and refining the microstructure. However, the large-scale industrial applications of Mg alloys containing these REs have been limited for the high price and resource issues. Hence, RE-free wrought Mg alloys with high mechanical properties should be developed. 

Mg-alkali alloys are currently attracting considerable attention due to their excellent performance. For example, Mg-Sr alloys which have been widely studied have shown great potential in the development of low-cost and high-performance Mg alloys. Researches have indicated that Sr can improve the ductility and the tension–compression yield asymmetry of Mg alloy [15,16]. However, the application of Mg-Sr binary alloy is limited due to their low mechanical properties. Recently, Mn is considered good alloying element to improve the mechanical properties of Mg-Sr binary alloy. Firstly, the existence of a high density of fine Mn precipitates during extrusion could evidently refine the grains of Mg alloys [17]. Secondly, the presence of un-dynamic recrystallized (un-DRXed) grain regions were generated in Mg alloys containing Mn, which has been proved to be helpful to improve the strength of the alloys [18,19]. Moreover, Mn element is a low-cost alloying element. Thus, Mg-Mn-Sr is a designed high strength and ductility Mg alloy [20,21,22,23,24,25,26]. Borar et al. had done many studies on the effect of Sr on the mechanical properties of Mg-Mn-Sr alloy, and they found that the strength and ductility of Mg-Mn-Sr alloy improved with the addition of Sr, but the tension–compression yield asymmetry of Mg-Mn-Sr alloy decreased [22]. They also clarified that the texture of Mg-Mn-Sr alloy was weakened during extrusion at 300 °C, which was caused by random orientation caused by continuous dynamic recrystallization (CDRX) [20]. Celikin et al. also investigated the microstructure and the creep behavior of Mg-Sr-Mn alloy and proved that the creep behavior was caused by the precipitation of Mn phase at the grain boundary during the creep process [25]. 

However, these researches are mainly focused on the effect of Sr on the microstructure and creep behavior of Mg-Mn-Sr alloys, and there are few studies about the effect of Mn on the microstructure and mechanical properties of Mg-Mn-Sr alloys. This study is to reveal the role of high Mn content on the microstructure and mechanical properties of the extruded Mg-Sr alloy, which can develop Mg-Sr-Mn alloy with a fine-grained microstructure and good mechanical properties.

## 2. Experimental Procedure

In this paper, the nominal compositions of alloys are Mg-0.5Sr, Mg-0.5Sr-1Mn, and Mg-0.5Sr-2Mn (wt.%), respectively. The experimental alloys were prepared by commercially pure Mg (99.9 wt.%), Mg-3 wt.% Mn, and Mg-30 wt.% Sr master alloys (Hunan Rare Earth Metal Material Research Institute, Hunan, China). The alloying components were completely melted in a steel crucible under the mixture gas of CO_2_ and SF6 at ~720 °C. Subsequently, the Mg-Mn-Sr melt was poured into a steel mould with a diameter of 100 mm and 150 mm in height, which had been preheated at 320 °C. The compositions of the samples were measured by X-Ray Fluorescence (XRF), and showed in Table 1. The difference between nominal composition and chemical composition was caused by the burning loss of alloy elements.

The ingots were preheated at 300 °C, for 1 h, and then extruded at 300 °C by a XJ-500 Horizontal Extrusion Machine (Yuanchang Machinery, Wuxi, China). The rods were extruded with a diameter of 16 mm, corresponding to extrusion ratio of 25:1, extrusion speed of 1 mm/min, and air-cooled to room temperature. The size of the tensile sample used for the tensile test is 25 mm in gage length and 5 mm in gage diameter, and the compression sample size in the compression test is 12 mm in gage length and 8 mm in gage diameter. The mechanical properties were analyzed by SANSIUTM 5000 (Xinsansi, Shenzhen, China) with a strain rate of 1.0 × 10^−3^ s^−1^. The instruments used to observe the microstructure are optical microscope OM, ( ZEISS NEOPHOT 3, Jena, Germany), scanning electron microscope SEM, (JEOL JSM-7800F, Tokyo, Japan) with an energy dispersive X-ray spectrometer (EDS) detector, transmission electron microscope TEM, (Tecnai G2 F20 S-TWIN, FEI, Hillsboro, OR, USA). For OM and SEM observations, the experiment samples were first ground on SiC paper, then etched in alcohol nitrate. For TEM observation, the experiment samples were first cut into thin slices of 0.5 mm thickness and ground to 60 μm thickness on metallographic sandpaper, then thinned by argon ion beam. The precipitated phase was analyzed by X-ray diffractometer (XRD, D/Max 2500 PC, Rigaku, Tokyo, Japan). The results of EBSD were detected by SEM (JEOL JSM-7800F, Japan) equipped with Oxford Instrument Nordlys Nano EBSD detector, and processed by Aztec and channel 5 software (Oxford instruments, Oxford, UK). In addition, EBSD experiment was carried out at 20 kV, 14 mm working distance, 70° tilt, and 0.2–0.3 scanning step.

## 3. Results

### 3.1. Microstructures before Extrusion

**Figure 1:**
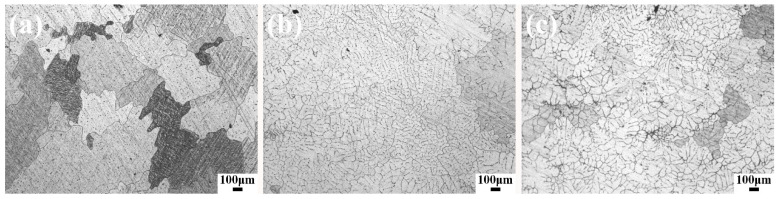
The optical microscope (OM) observations of as-cast alloys: (**a**) Mg-0.5 wt.% Sr, (**b**) Mg-0.5 wt.% Sr-1 wt.% Mn, (**c**) Mg-0.5 wt.% Sr-2 wt.% Mn.

Figure 1a–c shows the OM observations of the as-cast samples. The Mg-0.5Sr alloy (Figure 1a) consists of a coarse irregular grain structure. With Mn addition, the morphology of the Mg-Sr alloy becomes more dendritic. The grain size of the as-cast Mg-0.5Sr-xMn decreases gradually with the addition of Mn from 0 to 2 wt.%.

**Figure 2:**
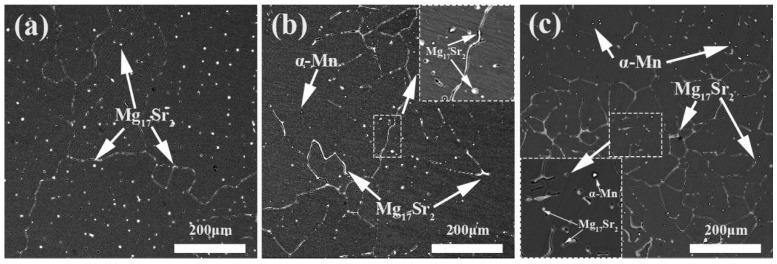
SEM-BSE micrographs of as-cast Mg-0.5Sr-xMn alloys: (**a**) x = 0 wt.%, (**b**) x = 1 wt.%, (**c**) x = 2 wt.%.

Figure 2 shows the SEM-BSE images of the as-cast Mg-0.5Sr-xMn alloys. The second phases were analyzed with EDS and the results were marked in the images. Mg17Sr2 particles and fine Mn particles are observed in Figure 2. And the Mg17Sr2 particles are distributed both at the grain boundaries and in the grains, while the Mn particles exist in the grains.

**Figure 3:**
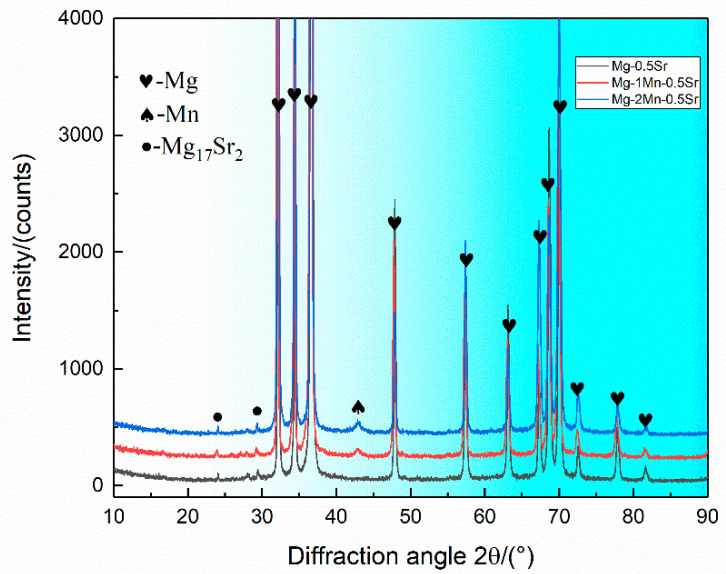
XRD patterns of as-extruded Mg-0.5Sr-xMn alloys, x = 0, 1, and 2 (wt.%).

Figure 3 shows the XRD results of the as-extruded Mg-Sr-xMn alloy. The results indicated that the Mg-Sr-Mn alloy is composed of α-Mg (matrix), α-Mn, and Mg17Sr2. And the existence of Mn phase was found in Mg-0.5Sr-1Mn and Mg-0.5Sr-2Mn alloys.

### 3.2. Grain Structure after Extrusion

**Figure 4:**
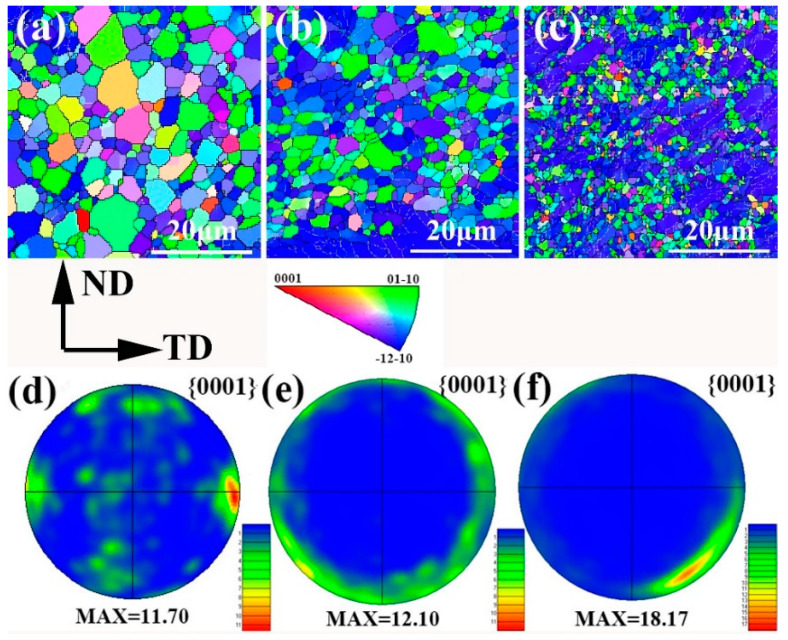
EBSD orientation maps and pole figures of as-extruded Mg-0.5Sr-xMn alloys: (**a**,**d**) x = 0 wt.%, (**b**,**e**) x = 1 wt.%, (**c**,**f**) x = 2 wt.%.

Figure 4 shows the inverse pole figures (IPF) maps and {0001} pole figure of the as-extruded Mg-0.5Sr-xMn alloys, which is perpendicular to the extrusion direction (ED). The average grain size of the as-extruded Mg-0.5Sr-xMn alloys is refined apparently by the addition of Mn. The grain size decreases from 2.78 µm to 1.15 µm with Mn addition. The IPF images indicate that the microstructures of Mg-0.5Sr-1Mn and Mg-0.5Sr-2Mn alloys consist of DRXed grain structures and un-DRXed grain structures. The structure of un-DRXed grains is dominated by coarse grains which is colored by blue in Figure 4c. The pole figures reveal that Mg-Sr alloy exhibits similar texture to the Mg-RE alloys, which is basal plane parallel to the ED. And Mg-0.5Sr-1Mn and Mg-0.5Sr-2Mn alloys exhibit the typical fiber texture. In addition, the intensity of {0001} texture in Mg-0.5Sr-2Mn alloy is largest among the Mg-0.5Sr-xMn alloys.

### 3.3. Second Phase

**Figure 5:**
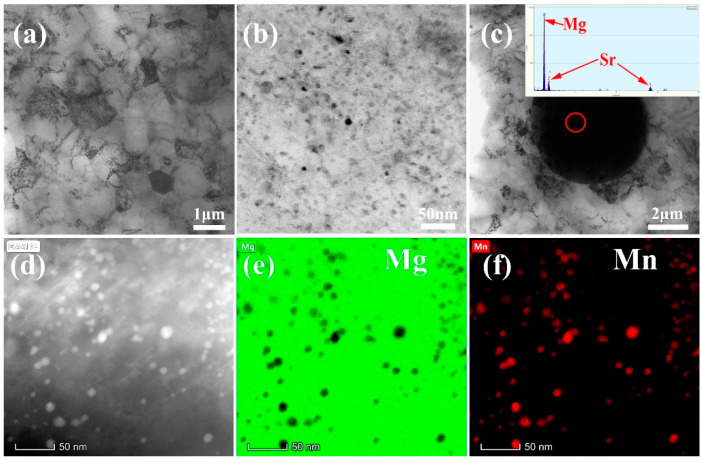
TEM micrographs of the of the as-extruded Mg-0.5 wt.% Sr-2 wt.% Mn alloy. (**a**) and (**b**) Bright field (BF) image; (**c**) BF and EDS results of second phase Mg17Sr2; (**d**–**f**) High Angle Annular Dark Field Imaging (HAADF) with EDS mapping.

The TEM results of the as-extruded Mg-0.5Sr-2Mn alloy is shown in Figure 5. The bright field (BF) image (Figure 5a) presents that the grain size is very small and nearly 1 μm. This result is the same as the above EBSD result. Several second phases are uniformly distributed in the Mg matrix, as shown in the BF image (Figure 5b). And it’s found that the size of Mg17Sr2 phase is about 5 μm, as shown in Figure 5c. The results of high-angle annular dark field (HADDF) and EDS mapping are shown in Figure 5d–f. The green color represents the distribution of Mg element and the red color represents the distribution of Mn element. It can be seen that the spherical-shaped precipitates are α-Mn phase.

### 3.4. Mechanical Properties

**Figure 6:**
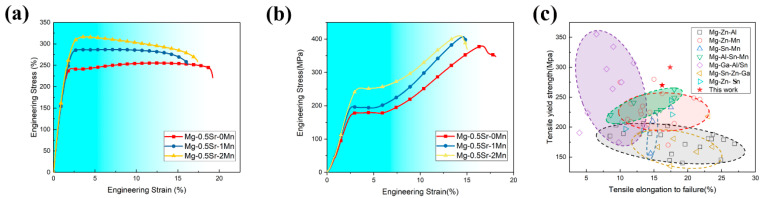
Effect of Mn on mechanical properties of as-extruded Mg-0.5Sr-xMn alloys in tensile testing along the extrusion (x = 0, 1, and 2 wt.%). (**a**) Engineering tensile stress-strain curves; (**b**) Engineering compression stress-strain curves; (**c**) Comparison of tensile yield strength (TYS) vs. strain for Mg-XMn-0.5Sr alloys with some common low-cost alloys [27,28,29,30,31,32,33,34,35,36].

Figure 6 shows the engineering stress-strain curves of the as-extruded Mg-0.5Sr-xMn alloys. The mechanical properties are shown in Table 2. As shown in Figure 6a,b, with the Mn addition, tensile yield strength (TYS) increases from 228 MPa to 300 MPa and ultimate tensile strength (UTS) increases from 255 MPa to 316 MPa. The elongation of alloys decreases from 19.68 to 17.39%. Compression yield strength (CYS) increases from 180 MPa to 252 MPa. The results show that the CYS of all alloys is significantly lower than the TYS. The value of CYS/TYS decreases from 0.79 (Mg-0.5Sr) to 0.71 (Mg-0.5Sr-1Mn), and then increases to 0.84 (Mg-0.5Sr-2Mn).

Compared with the conventional low-cost alloys, the TYS and EL of Mg-0.5Sr-xMn alloys are shown in Figure 6c. The ductility of Mg-0.5Sr-xMn alloys is higher than Mg-Ga-Al/Sn alloys and the TYS of Mg-0.5Sr-xMn alloys is higher than Mg-Zn-Mn/Al/Sn alloys. In general, Mg-Sr-Mn alloy is a kind of potential low-cost and high-performance Mg alloys.

### 3.5. Work-Hardening Behavior

**Figure 7:**
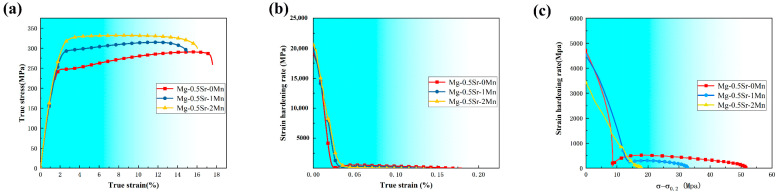
(**a**) Tensile flow curves of the tensile-loaded samples with different Mn; Work-hardening rate curves of the extruded Mg-Sn-Mn alloys: (**b**) θ vs. ε, (**c**) θ vs. (σ-σ0.2).

Figure 7a shows the true stress-strain curves of the extruded Mg-Sr-Mn alloys. The relevant analysis data of work hardening behavior are all from this curve. The strain hardening behavior of Mg-0.5Sr-xMn samples were analyzed by means of the strain hardening rate θ, defined as [37]:

(1) θ=dσ/dε

where σ and ε are true stress and plastic strain, respectively. Figure 7b shows the work-hardening rate vs. true plastic strain curves and Figure 7c shows the work-hardening rate vs. (σ-σ0.2) curves of the extruded Mg-0.5Sr-xMn alloys. In the beginning of work hardening, because of a short elastic-plastic transition, the work-hardening rates of all the samples decrease sharply with the increase of strain, which corresponds to stage I of work hardening. Secondly, the work-hardening rate decreases almost linearly with the increase of strain, corresponding to stage III. However, stage II does not exist in experiment alloys, which means a horizontal line exists between stage I and stage III on curves. Thus, according to the results, the work-hardening rate increases with the increment of Mn content during stage I. In contrast, the work-hardening rate decreases with the increase of Mn content during stage III.

**Figure 8:**
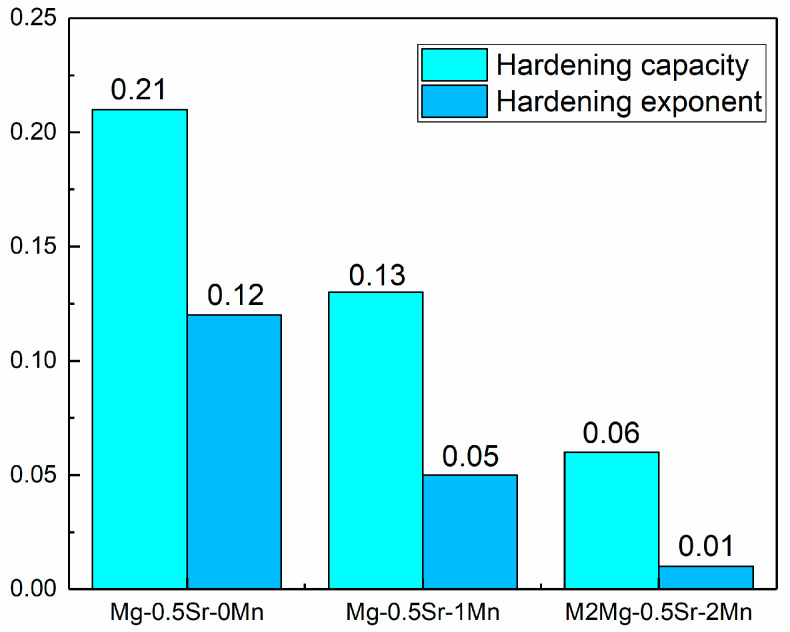
Hardening capacity and hardening exponent plots of the as-extruded Mg-Sr-Mn alloys.

The hardening capacity Hc of the alloys is defined as follows [38]:

(2) $$H_{c}=\left(\sigma_{UTS}-\sigma_{0.2}\right)/\sigma_{0.2}$$

where $\sigma_{UTS}$ is ultimate tensile strength, and $\sigma_{0.2}$ is the tensile yield strength. The hardening capacities of all the alloys are shown in Figure 8. It can be seen that the value of Hc decreases from 0.21 to 0.06 with the increase of Mn content from 0 to 2 wt.%.

The hardening exponent is determined as follows [39]:

(3) $$\sigma =K\varepsilon^{n}$$

where n is the hardening exponent and K is constant; the values for all the samples are given in Figure 8. Hardening exponent is an important parameter used to evaluate the formability of materials [40]. The value of n decreases from 0.12 to 0.01 with the increase of Mn content from 0 to 2 wt.%.

## 4. Discussion

### 4.1. Microstructures and Texture 

The grain size of as-cast Mg-0.5Sr–xMn decreases obviously with the addition of Mn, as shown in Figure 1, and the structure changes from irregular to dendrite. In this investigation, a number of second phases are observed in as-cast alloys (See Figure 2). The Mg17Sr2 and Mn precipitates can inhibit the growth of grain and contribute to form fine grain. In general, the fine grain of as-cast Mg-0.5Sr alloy is attributed to the increase of Mn content. 

The microstructure and texture can be determined by several factors during extrusion. Grain boundary bulging, dynamic recrystallization (DRX), particle stimulated nucleation (PSN), and grain growth after extrusion affect the microstructure and texture [41,42,43].

**Figure 9:**
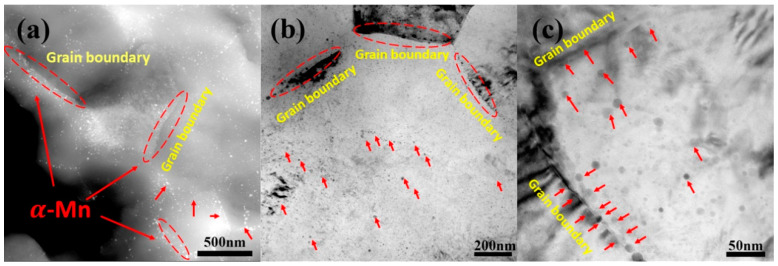
(**a**) S-TEM image of Mg-2 wt.% Mn-0.5 wt.% Sr alloy; (**b**) and (**c**) Bright field TEM images of Mg-2 wt.% Mn-0.5 wt.% Sr alloy. α-Mn are marked by red arrows and ellipses, and the Grain boundary are marked by yellow font. (**c**) is a partial enlarged view of (**b**), representing a complete grain.

Figure 4a–c showed that the grain size of as-extruded Mg-0.5Sr–xMn decreases with the addition of Mn. Mg-0.5Sr-2Mn alloy shows bimodal structures (DRXed and un-DRXed grain structures), while Mg-0.5Sr and Mg-0.5Sr-1Mn alloys exhibit DRXed structures. Previous studies have shown that the existence of particles precipitated during extrusion can refine grains in Mg-Sr-based alloys [41], which also existed in Mg-Al-Mn alloys [42]. In this work, the presence of fine Mn particles can restrain grain growth and form fine DRXed grain structures during extrusion. At the same time, un-DRXed grains were formed in Mg-Sr alloys with high Mn content. The fine structure is due to many grain boundaries and second particles served as the nucleation sites during recrystallized through DRX and PSN mechanisms. It is reported that the fine precipitated phase can pin the recrystallized grain boundary and the fine recrystallized grains are preserved. On the other hand, stacking of dislocations closed to the grain boundary can also cause the nucleation of recrystallized grains. In this work, a large amount of Mn exists near the grain boundary in the as-extruded Mg-0.5 Sr–2 Mn alloy, as shown in Figure 9. It can be seen that nanoscale Mn precipitates distribute along the grain boundaries or in the grain interiors. They exhibit strong pinning effect and suppress the growth of the recrystallized grains. Thus, refined grains were generated. Moreover, many tiny Mg17Sr2 precipitates lay in the interior of the grains and along the grain boundary in as-cast experiment alloys, as shown in Figure 2. They are the nucleation sites that induce the nucleation of the recrystallized grains through PSN mechanism during extrusion. Generally, under the two mechanics, the fine recrystallized grains were obtained after extrusion.

The PSN mechanism plays a vital role in the texture intensity of the recrystallized grains [43,44]. Due to the PSN mechanism, the fine grain and random texture, which were different from the original crystal grains, were obtained. The Mg-0.5Sr alloy has a similar texture to the Mg-RE alloys, so, the orientation is more random than Mg-0.5Sr alloy. The Mg-0.5Sr-1Mn and Mg-0.5Sr-2Mn alloys show a typical fiber texture after extrusion and have higher intensity of texture than the Mg-0.5Sr alloy. In general, fine DRXed grains can weaken the intensity of texture, while coarse un-DRXed grains generally exhibit strong basal texture. With the content of Mn increases, the number of un-DRXed grains increases, so, the intensity of basal texture increases.

### 4.2. Mechanical Properties

The mechanical properties of as-extruded alloys are mainly affected by the grain size, precipitates, and the intensity of texture. Grain boundaries can hinder dislocation slip and twin growth. Grain refinement means that the total area of grain boundaries increases, thus, enhancing the yield strength. In this paper, the effect of grain size on yield strength is explained by the Hall–Petch (HP) equation [45]:

(4) $$\sigma_{\mathrm{gs}}=kd^{-1/2}$$

where $\sigma_{\mathrm{gs}}$ is the strength contribution from grain boundaries, k is the coefficient of HP related to the alloys, and d is the grain size. Researches have indicated that the parameters of the tensile yield strength depend on the grain size and texture. And due to the high k coefficient, Mg alloys have more pronounced hardening behavior than that of Al alloys [46,47]. Yuan et al. [48] found that k is 205 MPa μm^1/2^ when the grain size is about 2 μm. In this work, the grain size and texture are similar to Yuan’s work. So, ignoring the difference in solute strengthening between AZ31 and the Mg-Sr-Mn alloy, the above-mentioned value can be used to estimate the grain refinement and hardening effect. Therefore, the strength contribution from grain boundaries of the Mg-0.5Sr-xMn (x = 0, 1, and 2 wt.%) alloys is 125 MPa, 141 MPa, and 191 MPa, respectively. 

The other important factor is the precipitated phase, which also affects the TYS of the experiment alloys. In general, the uniform distribution of the fine second phase in the magnesium matrix is good to mechanical performance [37]. Figure 9 shows that the Mg-0.5Sr-2Mn alloy have many nanoscale α-Mn particles dispersed in the matrix, which can effectively impede the movement of dislocations. In general, the interaction between the second phases and dislocations can be quantitatively assessed by Orowan relationship [49] as follows:

(5) $$\sigma_{ps}=M\frac{Gb}{2\pi \lambda \sqrt{1-\upsilon }}\ln\left(\frac{D_{p}}{r_{0}}\right)$$

where $\sigma_{ps}$ is the yield strength of precipitation strengthening, M is the Taylor factor (M = 4.5), G is the shear modulus of Mg matrix (G = 1.66 × 10^4^ MPa), b is the Burgers vector of gliding dislocations (b = 3.21 × 10^−10^ m), λ is the effective inter-particle spacing of α-Mn, υ is the Poison ratio (υ = 0.29), $D_{p}$ is the mean diameter of precipitated particle, $r_{0}$ is the core radius of the dislocation, which is usually considered to be $r_{0}$ = b. Thus, according to Orowan relationship, the second phase strengthening is calculated to be 34 MPa.

Texture is also a vital factor to affect the mechanical properties of Mg alloys. In Figure 4f, the Mg-0.5 wt.% Sr-2 wt.% Mn alloys shows the highest intensity of texture, which is unfavorable to activation of basal dislocation slip, thus, promoting the improvement of the strength. In this work, combined with the effect of grain size and intensity of texture on the strength can be explained by follows [50]:

(6) $$\sigma_{g-t}=\frac{0.3}{m_{t}}\sigma_{g}$$

where $\sigma_{g-t}$ is the yield strength including the effect of grain size and the intensity of texture, $m_{t}$ is the average Schmid factor, and $\sigma_{g}$ is the strength contribution from the grain boundaries. According to the EBSD data results, the value of $\sigma_{g-t}$ for Mg-0.5Sr-2Mn alloy is calculated to be 220 MPa. 

The tensile and compression yield asymmetry (CYS/TYS) is often observed in Mg alloys. In this work, Mg-0.5Sr-1Mn and Mg-0.5Sr-2Mn alloys have the fiber texture. And {101(−)2} extension twinning can occur during compression along the ED. It can be seen from Figure 4, the addition of Mn improves the texture intensity due to the volume of the un-DRXed grains increasing. Twinning is more likely to occur in un-DRXed grains, which improves the tension-compression asymmetry [51]. Also, the reduction of grain size has a greater influence on CYS than TYS, which decreases the yield asymmetry. Therefore, under the combined action of grain size and the number of un-DRXed grains, the CYS/TYS of Mg-0.5Sr-xMn alloys decrease from 0.79 to 0.71, and then increase from 0.71 to 0.84.

### 4.3. Work-Hardening Behavior

At room temperature, Mg alloys have limited slip systems due to their hcp structure, which causes the strain hardening behavior of Mg alloys to be different from that of cubic metals. It is reported that the strain hardening behavior dominated by dislocation slip and twinning can be greatly influenced by texture, solid solution element, and grain size [52]. In the present study, the solid solution element is negligible because Mn has low solubility at room temperature. So, the effect of texture and grain size on the strain hardening behavior are mainly discussed in this section.

From the above, it’s known that the texture affects the deformation behavior of Mg alloys. Previous studies [53] have reported that {101(−)2} twinning has significant influence on the tensile deformation when the tensile axis is 0° to the c-axis of the grain. The basal slip becomes its dominant deformation mechanism when the tensile axis is 45° to the c-axis of the grain. The basal slip and {101(−)2} twins are difficult to activate when the tensile axis is 90° to the c-axis of the grain and the deformation mechanism is mainly dominated by prismatic slip. Mg-0.5Sr alloy has a weaker texture intensity as compared with the alloys containing Mn. The reason is the DRX mechanism. So, the strain hardening behavior of Mg-0.5Sr alloy can be divided into three stages, shown in Figure 7. In stage I or the elastoplastic transformation stage, {101(−)2} initiation of tensile twins results in macroscopic yielding. In stage II, {101(−)2} nucleation and growth stages of twins occur. The initial strain hardening rate is low, but the strain hardening rate increases linearly as the strain increases. In stage III, {101(−)2} tensile twins are saturated and the strain hardening rate falls off gradually with the growth in strain. However, Mg-0.5Sr-1Mn and Mg-0.5Sr-2Mn alloys have high-intensity texture due to the existence of un-DRXed grains. Some grains are oriented in a hard orientation, which can hinder the initiation of basal dislocation slip and promote the activation of twins to coordinate the plastic deformation. So, the strain hardening behaviors of Mg-0.5Sr-1Mn and Mg-0.5Sr-2Mn alloys change from stage I to stage III directly.

Valle et al. [53] reported that grain size affects the work-hardening behavior. For Mg-0.5Sr-1Mn and Mg-0.5Sr-2Mn alloys, linear hardening stage strings along the dramatic decrease in strain hardening rate stage (i.e., a unique slip deformation behavior, in which the grain size and dynamic recovery significantly affect the work hardening behavior [53]). In other words, for equiaxed small grains of uniform size, dislocations cannot be readily trapped inside because it is easy to reintegrate into the grain boundaries from all directions within a short distance. Thus, many dislocations which accumulated at the grain boundaries lead to stress concentration at the grain boundaries. Therefore, most stresses are due to reorganization and annihilation inside the boundaries. In a word, with the grain size decreases, most stresses can easily balance out through non-basal slip, dynamic recovery, and grain boundary sliding during the plastic deformation process. In this case, the contribution of twinning reduces, and the pronounced work hardening cannot be sustained. Similarly, we can explain the phenomenon based on the work hardening rate in stage III. The $\;\theta_{\mathrm{III}}$ represents the work-hardening rate during stage III and it is obtained by extrapolating to ($\sigma -\sigma_{0.2}$) = 0 (shown in Figure 7c). The values of $\theta_{\mathrm{III}}$ of Mg-0.5Sr, Mg-0.5Sr-1Mn, and Mg-0.5Sr-2Mn alloys are 852, 629, and 435 MPa, respectively. The result shows that $\theta_{\mathrm{III}}$ decreases with Mn addition because of the grain refinement. In addition, strong basal texture can also decrease the work hardening rate. Liao et al. [54] have also reported that the increase of Mn content leads to decrease in the work hardening rate in Mg alloys.

## 5. Conclusions

High yield strength and ductility in as-extruded Mg-0.5Sr alloy were achieved by high Mn alloying in this study. The major conclusions are summarized as follows:

1. The grain size of as-extruded alloys is refined by the addition of Mn. The main reason is that the growth of recrystallization grains is suppressed by the nanoscale Mn precipitates during extrusion.
2. The main factors for the improvement in the TYS and CYS of Mg-0.5Sr-xMn alloys are refined microstructure, strengthened texture, and large volume of nanoscale Mn precipitates.
3. Mn can significantly reduce the work hardening behavior of Mg-Sr alloy. With the addition of Mn, the values of Hc and n significantly decreased. The decrease in the alloy’s work-hardening ability is mainly due to grain refinement by addition of Mn.
4. Mg-0.5Sr alloy with Mn addition has fine microstructures and good mechanical properties, which is a potential low-cost and high-performance magnesium alloy.

## Figures and Tables

**Table 1:** The chemical compositions of Mg-0.5Sr-xMn alloys (wt.%).

Alloys	Sr	Mn	Mg
Mg-0.5Sr	0.15	0	Bulk
Mg-0.5Sr-1Mn	0.18	0.94	Bulk
Mg-0.5Sr-2Mn	0.14	1.98	Bulk

**Table 2:** Mechanical properties of as-extruded Mg-0.5Sr-xMn alloys with different Mn (x = 0, 1, and 2 wt.%).

Alloys	TYS (MPa)	UTS (MPa)	EL (%)	CYS (MPa)	CYS/TYS
Mg-0.5Sr	$228_{-2}^{+3}$	$255_{-4}^{+6}$	$19.6_{-0.3}^{+0.2}$	$180_{-4}^{+3}$	0.79
Mg-0.5Sr-1Mn	$270_{-4}^{+2}$	$286_{-3}^{+5}$	$16.2_{-0.3}^{+0.8}$	$193_{-3}^{+6}$	0.71
Mg-0.5Sr-2Mn	$300_{-2}^{+5}$	$316_{-2}^{+4}$	$17.3_{-0.6}^{+1.1}$	$252_{-4}^{+5}$	0.84
